# 750. Retrospective Evaluation of the Three-Step EIA/PCR Algorithm as a Cost-Effective Method for Detection and Treatment of Clostridium Difficile Infection

**DOI:** 10.1093/ofid/ofab466.947

**Published:** 2021-12-04

**Authors:** Bakri Kulla, patrick Haggerty

**Affiliations:** EVMS, Norfolk, Virginia

## Abstract

**Background:**

*Clostridium difficile* infection (CDI) is the primary cause of infectious diarrhea in the United States. With an estimated 453,000-500,000 burden cases that are associated with 15,000-30,000 deaths annually in the United States. Because of its prevalence, there is a projected 3.2-4.8 billion dollar annual cost for inpatient care related to CDI. For these reasons, accurate and timely detection of CDI is crucial to reduce the morbidity, mortality, and medical costs.

**Methods:**

This is a retrospective cohort study. Adult patients, aged 18 through 80 years, admitted between 9/1/2016 and 9/30/2017, who presented with diarrhea and received a CDI algorithm test. To assess bivariate associations between true positive and indeterminate positive groups, categorical variables were compared using Chi-Square or Fisher’s exact tests when appropriate, and continuous variables were analyzed using independent samples t-tests.

**Results:**

The study included 1031 stool samples, of which 853 (82.7%) were CDI negative and 178 (17.3%) were CDI positive. Of the full sample, 265 (25.7%) were GDH (+), 94 (9.1%) were toxin (+), and 84 (8.1%) were PCR (+).

In order to examine patient-level variables, the first positive from each patient was included to ensure independence of data points, resulting in 830 unique tests and patients. The true positive rate of this sub-sample was 9.4% (*n* = 78) and indeterminate positive rate was 8.7% (*n* = 72).

An important findings of the study is that of the patients who were GDH (+)/toxin (-), 87 (50.9%) were PCR (-) and 84 (49.1%) were PCR (+).Table 1

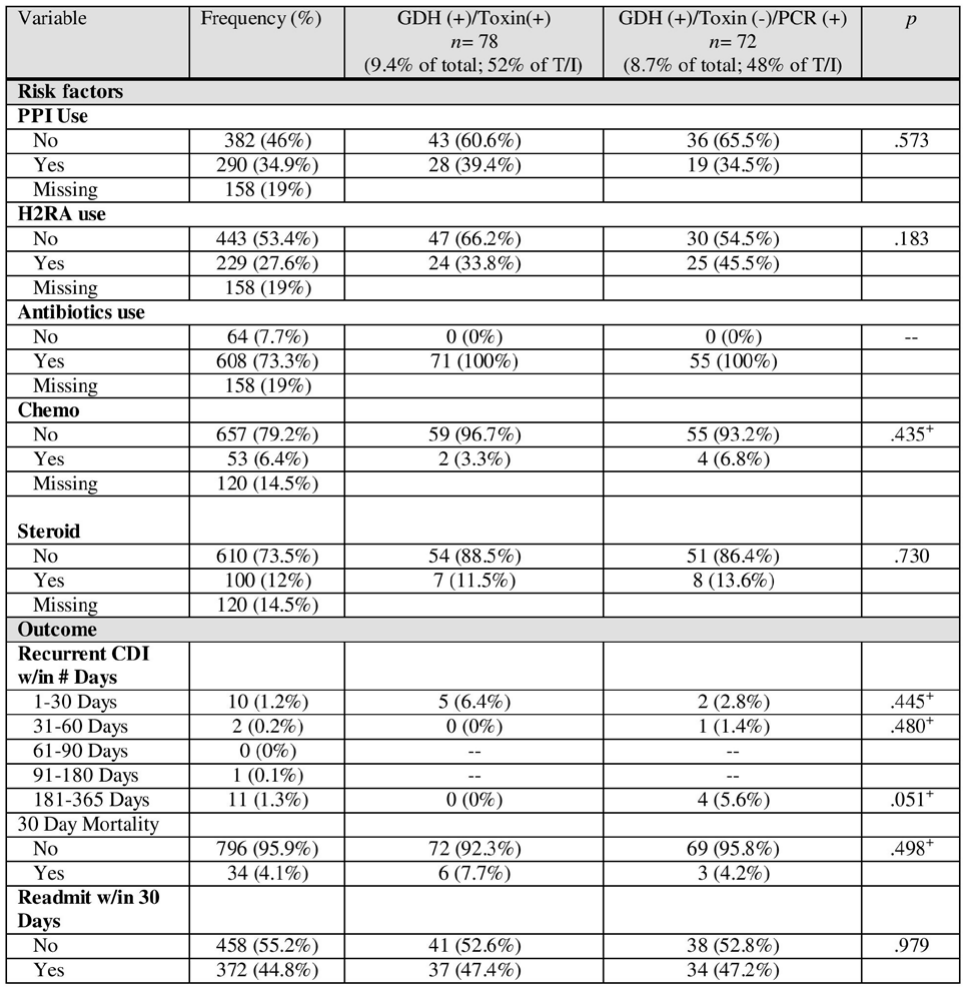

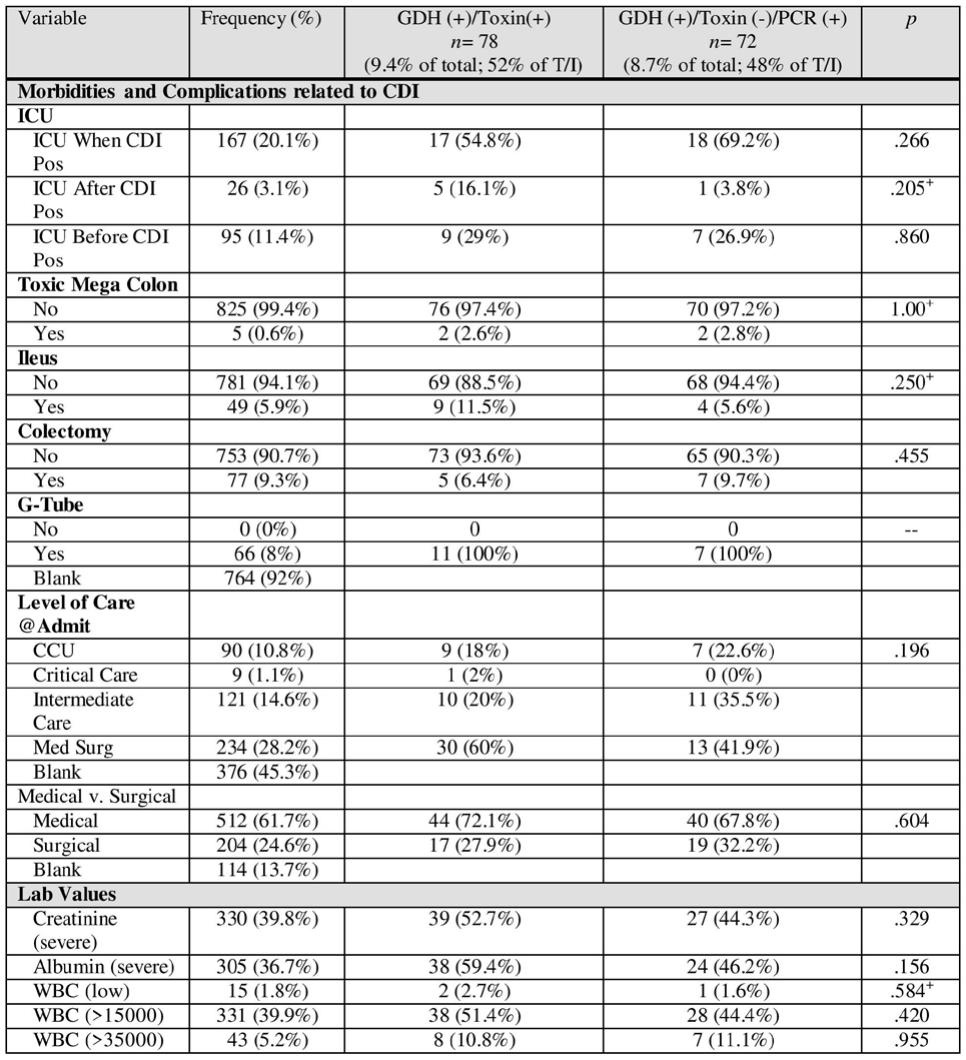

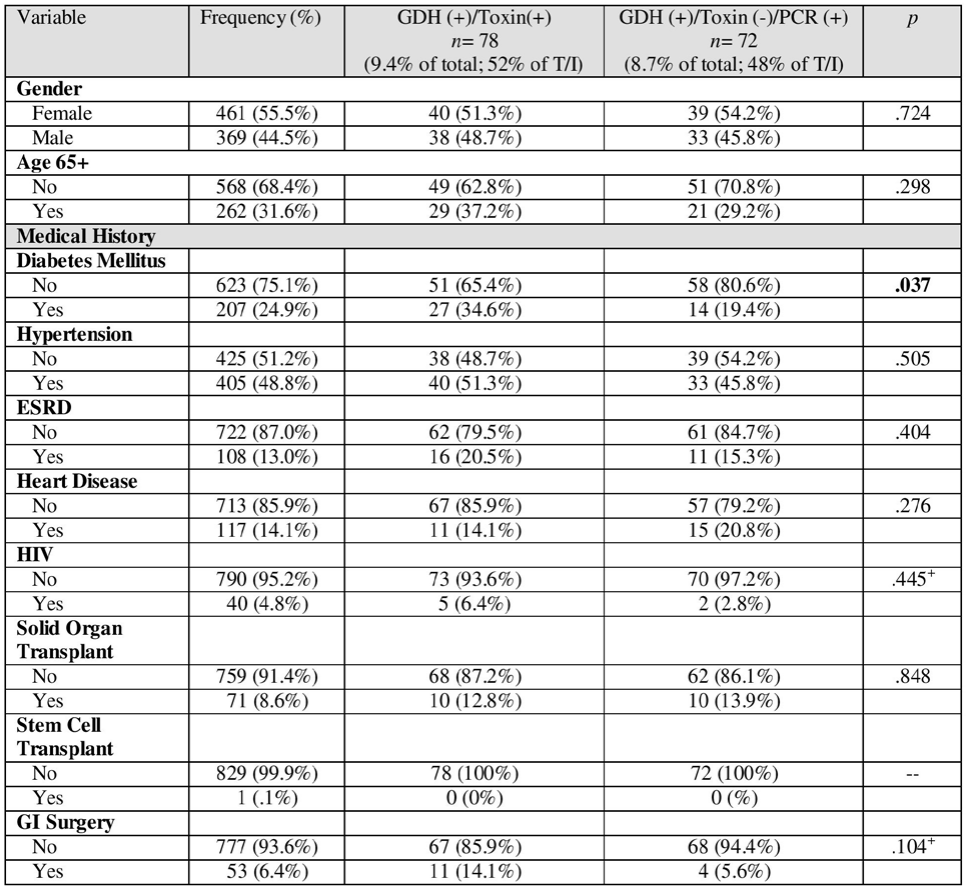

**Conclusion:**

The study found that of the patients who are GDH (+) and Toxin (-), the PCR test serves as a proxy for the CDI test. In addition, we demonstrated that whether the patient was true positive by the GDH/Toxin test or indeterminate positive, the outcomes were the same. The only difference was the antibiotic selections for treatment. Performing PCR tests as a part of three-step algorithm prevented nearly half of discrepant patients from being unnecessarily treated with antibiotics and placed on enteric precaution, thereby extending their hospital stay. Finally, by preventing unnecessary antibiotic use, isolation and hospital length of stay, it is proposed that the three-step algorithm effectively reduces hospital cost.

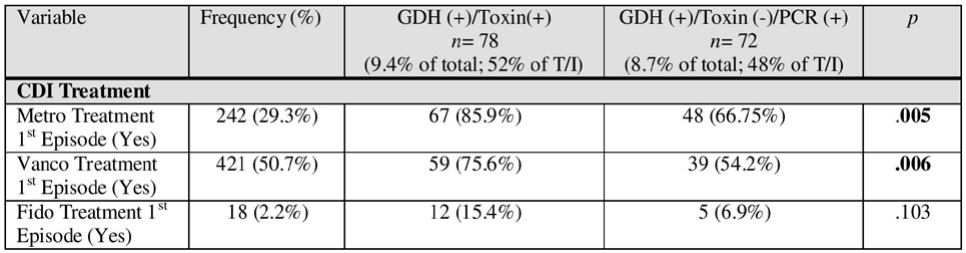

**Disclosures:**

**All Authors**: No reported disclosures

